# Understanding Inflammasomes and PD-1/PD-L1 Crosstalk to Improve Cancer Treatment Efficiency

**DOI:** 10.3390/cancers12123550

**Published:** 2020-11-27

**Authors:** Anaïs Perrichet, François Ghiringhelli, Cédric Rébé

**Affiliations:** Platform of Transfer in Cancer Biology, Centre Georges François Leclerc, INSERM LNC UMR1231, University of Bourgogne Franche-Comté, F-21000 Dijon, France; aperrichet@cgfl.fr (A.P.); fghiringhelli@cgfl.fr (F.G.)

**Keywords:** cancer, inflammasomes, interleukin, immune checkpoint, PD-1, PD-L1

## Abstract

**Simple Summary:**

Immune checkpoints and inflammasomes have been shown to regulate cancer progression and response to treatments. This review summarizes the recent findings on the crosstalk between the immune checkpoint PD-1 and inflammasomes and the promising association of treatments that might be efficient for cancer patients.

**Abstract:**

Inflammasomes and immune checkpoints have been shown to participate in carcinogenesis, cancer growth and response to treatment. Thus, targeting cytokines resulting from inflammasome activation, such as interleukin (IL)-1β, has emerged as a new tool in the therapeutic arsenal. Moreover, the use of checkpoint inhibitors such as anti-PD-1 or anti-PD-L1 has revolutionized the treatment of some cancer patients. However, inflammasome activation and consecutive cytokine release only occurs in some chemotherapeutic treatments and immune checkpoint inhibitors only work for a restricted number of patients, thus limiting the use of therapies targeting these pathways. Expanding knowledge about the inefficiency of these therapies recently brought forward the hypothesis of targeting both pathways. In this review, we provide an overview of the crosstalk between inflammasomes and programmed death-1 (PD-1)/programmed death-ligand 1 (PD-L1) that might explain how these two pathways are mutually dependent, and perhaps why targeting only one of them leads to inefficiency of cancer treatment in some patients.

## 1. Introduction

The body can sense physiological and pathogenic inflammatory stimuli, including through inflammasomes. These multi-proteic complexes are part of the innate immune response and participate in the clearance of damaged cells or pathogens, respectively called damage-associated molecular patterns (DAMPs) and pathogen-associated molecular patterns (PAMPs). This innate immune response then allows T-dependent adaptive immune response to be established. Adaptive response requires antigen processing and presentation, in the presence of inflammatory signals, to transiently recruit and activate antigen-specific immune cells, such as dendritic cells. The crosstalk between the innate and adaptive immune systems is necessary for the finetuning of the adaptive immune response, and inflammation is essential to control activation or inhibition of the immune response.

Adaptive and innate responses are involved in cancer emergence, progression and response to conventional treatments. One of the possible impediments to cancer immune response is the crosstalk between innate and adaptive immune response. We will focus here on two main families involved in innate and adaptive immune response, i.e., respectively inflammasomes and immune checkpoints PD-1/PD-L1, to understand how they mutually regulate each other to foster or dampen antitumor immune response.

### 1.1. Inflammasomes

**Figure 1:**
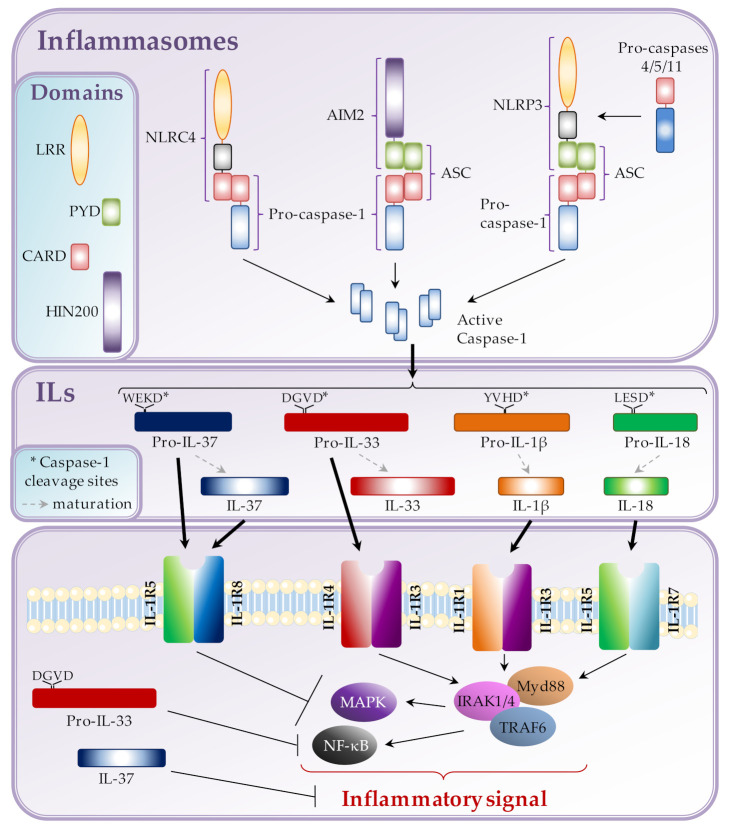
Inflammasome assembly and caspase-1 activation leads to the cleavage of specific cytokines to regulate inflammatory response.

Inflammasomes are subdivided into three main families, the nucleotide-binding domain (NOD)-like receptors (NLRs), absent in melanoma 2 (AIM2)-like receptors (ALRs) and pyrin. These families are characterized by specific domains that compose the inflammasome receptor. Their activation, via host danger, viral or bacterial signals, leads to the recruitment of inflammatory caspases, sometimes via their association with adaptor proteins. When activated caspases trigger the cleavage of pro-interleukins into mature interleukins (Figure 1).

#### 1.1.1. NLR Family

The NLR family is composed of the NLRA, NLRB, NLRC and NLRP sub-families. These receptors have a central nucleotide-binding domain (NBD), and most of them are composed of a C-terminal leucine-rich repeat (LRR). However, only NLRC and NLRP members contain a caspase activation and recruitment domain (CARD) and/or a pyrin domain (PYD) to recruit and activate caspases [1].

NLRC1, 2, 3 and 5 have regulatory functions on several pathways, such as transcription or kinase activation. NLRC4 is recruited by NAIPs (neuronal apoptosis inhibitor protein) after detection of bacterial flagellin or type II secretion system components [1]. NLRC4 contains a CARD, which enables the formation of a functional inflammasome, by recruiting caspase-1 (through CARD interaction). Its activation leads to production of IL-1β and IL-18 [2].

The NLRP sub-family is composed of 14 members. NLRP4, 5, 8, 9, 10, 11, 13 and 14 cannot form a functional inflammasome, while NLRP1, 2, 3, 6, 7 and 12 can. These NLRPs were shown to interact with apoptosis-associated speck-like protein containing a CARD (ASC), and NLRP1, which possesses a CARD, can directly recruit procaspase-1. All these NLRPs recognize microbial agents [3,4,5,6,7]. NLRP3 inflammasome is the most widely studied complex, perhaps due to its capacity to be activated by many signals and its involvement in numerous pathologies. The expression of NLRP3 must be up-regulated through nuclear factor kappa-light-chain-enhancer of activated B-cells (NF-κB) activation, in a first step called “priming”. A variety of stimuli able to activate NLRP3 trigger three non-exclusive molecular pathways that can interact with each other [8,9]. The first pathway consists in ion efflux, e.g., intracellular K^+^ efflux induced by extra-cellular ATP binding on its receptor P2RX7. The second pathway, lysosome damage, can be induced by crystalline structure phagocytosis. Lysosomal content released in the cytosol, especially cathepsin B, activates NLRP3 through a direct interaction. The third pathway is an increase in ROS synthesis. All these pathways lead to NLRP3 activation, recruitment of ASC and pro-caspase-1, and ultimately to IL-1β and IL-18 maturation and secretion [8,9].

#### 1.1.2. ALR Family

The ALR family members are constituted by an N-terminal PYD and a C-terminal hematopoietic interferon-inducible nuclear protein with 200-amino acid repeat (HIN200) domain. Absent in melanoma 2 (AIM2) is the most widely studied member, and the function of other members is not clear. In the absence of stimuli, PYD and HIN200 domains are associated. AIM2 senses DNA (cytosolic dsDNA of viral or bacterial origin or DNA from dying cells) through its HIN200 domain, thus rendering the PYD available for further interaction with ASC and subsequent caspase-1 activation [10].

#### 1.1.3. Non-Canonical Inflammasomes

Apart from caspase-1, the inflammatory caspase family is also composed of murine caspase-11 and its human homologs caspase-4 and 5. These caspases are activated by intracellular LPS, through direct interaction, then forming a non-canonical inflammasome. Once activated, this atypical inflammasome induces caspase-1 activation, which in turn cleaves pro-IL-1β and pro-IL-18 and also induces GSDMD proteolytic processing to generate cell membrane pores and pyroptotic cell death [11].

### 1.2. Inflammasome Processed Cytokines

As seen above, the major role of inflammasomes is to process pro-cytokines into mature cytokines which are then secreted in the extracellular environment. Once released, these cytokines bind to their receptors, in an autocrine and/or paracrine manner and activate intracellular molecular signaling pathways. Inflammasome-mediated IL-1β, and to a lesser extent, IL-18 maturation have been well described. However, inflammasomes are also able to mature other cytokines of the IL-1 family, such as IL-33 or IL-37 (Figure 1).

#### 1.2.1. IL-1β

Transcription of the Il1b/IL1B gene depends on many transcription factors. The most important is NF-κB, which is activated by ligands of toll-like-receptors (TLRs), namely lipopolysaccharides (LPS), but also by tumor necrosis factor (TNF)α, through the TNF receptor or IL-1β itself [12,13]. Other transcription factors are also involved in Il1b/IL1B transcription, such as hypoxia-induced factor 1 (HIF1), CCAAT/enhancer binding protein (C/EBP-)β, Interferon response factors 4 or 8 (IRF4/8) and PU.1, protein kinase C (PKC)/activator protein-1 (AP-1) or Signal transducer and activator of transcription (STAT)1 [12,14].

Pro-IL-1β is a 31kDa precursor protein containing 269 amino acids. A YVHD (Tyr-Val-His-Asp) domain enables caspase-1 cleavage after aspartic acid D116, leading to the maturation of the pro-form pro-IL-1β into the mature 17kDa active IL-1β [15,16].

IL-1β is mainly produced by myeloid cells, i.e., macrophages and dendritic cells. However, many recent studies have shown that other intra-tumor cells can produce IL-1β. Among these are myeloid-derived suppressor cells (MDSCs), fibroblasts or cancer cells themselves. Once secreted, IL-1β binds to IL-1 Receptor 1 (IL-1R1) and IL-1R3 (also known as IL-1RAcP) heterodimer, expressed at the cell surface of many cell types, such as epithelial or immune cells. Then, these receptors recruit intracellular molecular adapters and activate many transcription factors, such as activator protein-1 (AP-1) and NF-κB, to induce the transcription of target genes involved in several biological processes, depending on the cell type stimulated by IL-1β. This signal is also regulated by the expression of the decoy receptor IL-1R2 and by the production of the antagonist IL-1RA [17,18].

Usually, IL-1β has a pro-tumor role but in some cases, depending on the tumor type or the treatments used, it can act as an anti-tumor cytokine. Thus, therapeutic targeting of IL-1β can be used in some, but not all cases [18]. Many antibodies have been developed to block IL-1β. The IL-1RA anakinra is one of the most widely used in pre-clinical studies. Anakinra is a non-glycosylated form of human IL-1RA that competitively inhibits IL-1α and IL-1β from binding to their receptor. It has shown benefits in several clinical trials [19,20,21]. Canakinumab is a specific human monoclonal IgG1 antibody that targets IL-1β. This antibody has shown benefits for lung cancer incidence and patient mortality in a phase three clinical trial (CANTOS) [22].

#### 1.2.2. IL-18

Pro-IL18 is expressed constitutively in most cell types, including macrophages, DCs, fibroblasts or epithelial cells. It appears to be less tightly transcriptionally regulated than IL-1β, although its mRNA expression was shown to be up-regulated in inflammatory lesions [23].

Pro-IL-18 is a 24kDa precursor protein containing 193 amino acids. A LESD (Leu-Glu-Ser-Asp) domain enables caspase-1 cleavage after aspartic acid D36, leading to the maturation of pro-IL-18 into the mature, 17 kDa active form IL-18 [15,16].

Once secreted, IL-18 binds to IL-18Rα and IL-18Rβ heterodimer (IL-1R5/IL-1R7). Then, these receptors recruit intracellular molecular adapters such as Myd88 and activate transcription factors, such as AP-1 and NF-κB, to induce the transcription of target genes involved in several biological processes, depending on the cell type stimulated by IL-18 [17].

The role of IL-18 in cancer is unclear. In some cases, high levels of IL-18 have been associated with advanced tumor stage or poor prognosis [24], suggesting a pro-tumorigenic role for IL-18. However, IL-18 is also able to enhance IFN-γ production from Th1 cells, leading to natural killer (NK) and CD8^+^ T cell anti-tumor response [25]. Moreover, the importance of IL-18BP (IL-18 binding protein), an antagonist of IL-18, should also be considered when analyzing the effects of IL-18 in cancer [26].

In view of these ambiguous effects of IL-18, strategies aimed at increasing or blocking IL-18 were tested in preclinical models. Anti-IL-18 antibodies have shown interesting results in multiple myeloma [27], while IL-18 delivery using oncolytic viruses or bacteria delay melanoma, colon or breast cancer growth [28,29]. In humans, a phase 2 clinical study showed that recombinant IL-18 had limited activity in metastatic melanoma patients [30].

#### 1.2.3. IL-33

Pro-IL-33 is expressed by stromal, epithelial and hematopoietic cell types and has dual effects. Due to the presence of a nuclear localization signal (NLS), it is localized in the nucleus where it associates with chromatin. To the best of our knowledge, pro-IL-33 target genes have not yet been identified. However, its interaction with NF-κB inhibits NF-κB target gene expression [31]. Pro-IL-33 is also passively released in the extracellular space, mainly by dying cells. Then, pro-IL-33 binds to its receptor, IL-1R4 (or ST2) in association with IL-1RAcP, expressed on many lymphoid and myeloid cells, and activates signaling pathways dependent on NF-κB, c-Jun N-terminal kinase (JNK), and p38 mitogen activated protein kinase (MAPK) [32,33,34].

Pro-IL-33 is an active 31kDa protein containing 270 amino acids. The possibility for caspase-1 to cleave pro-IL-33 is a matter of debate. However, a DGVD (Asp-Gly-Val-Asp) domain was described to enable caspase-1 cleavage after aspartic acid D178, leading to the inhibition of IL-33 [15,16,35]. Contrary to IL-1β and IL-18, IL-33 cleavage by caspase-1 generates an inactive form. Thus, inflammasome-mediated IL-33 cleavage inhibits IL-33 function.

Like IL-18, IL-33 was shown to have pro- and anti-tumor effects. It seems that host cells producing IL-33, such as cancer-associated fibroblasts (CAFs), are responsible for tumor progression by inducing migration, epithelial–mesenchymal transition (EMT), invasion and proliferation of cancer cells, while in cancer cells, IL-33 is responsible for the improvement of anti-tumor immune response [33,34]. Although these dual effects may be explained by the localization of IL-33, it seems to be more complicated, since administration of recombinant IL-33 leads to both pro- and anti-tumor effects.

#### 1.2.4. IL-37

Five isoforms of pro-IL-37 (a–e) have been reported. While the c and e isoforms are known to be non-functional, pro-IL-37b is the most widely studied. It is expressed by monocytes, macrophages, B cells, plasma cells, T cells, neoplastic cells as well as epithelial cells. Its expression can be induced by IL-1β, TLR stimulation and TGFβ [32,33,36,37].

Pro-IL-37b is a 30kDa protein containing 218 amino acids. A WEKD (Trp-Glu-Lys-Asp) domain enables caspase-1 cleavage after aspartic acid D20 [38]. Like IL-33, IL-37 has a role in the nucleus and outside the cell. Caspase-1-cleaved IL-37 binds Smad3 in the cytosol, which carries it into the nucleus to increase non-receptor protein tyrosine phosphatases (PTPNs), thus promoting dephosphorylation of several tyrosine phosphorylation-dependent signaling pathways such as ERK, p38 MAPK, JNK, PI3K, NF-κB, and STAT3, leading to their inhibition [39]. In the extracellular space, pro-IL-37 and cleaved IL-37 both bind IL-1R5 (IL-18Rα) and IL-1R8 (also known as SIGIRR), thus not only inhibiting the association of IL-18 with its receptor, but also inducing an inhibitory signal. Thus, the dual role of IL-37 drives an anti-inflammatory response [32,33,36].

IL-37 has anti-tumor properties, through its capacity to inhibit inflammation and STAT3 or NF-κB activation. However, some pro-tumor effects have also been reported [33]. The capacity of IL-37 to bind IL-18Rα is not caspase-dependent. Caspase-1 cleavage of IL-37 seems only to affect its capacity to translocate into the nucleus. The generation of inhibitors targeting the IL-37/Smad3 interaction might be investigated to target effects mediated by cleaved IL-37.

### 1.3. The Immune Checkpoint Programmed Cell Death-1 Receptor (PD-1)

Tumor cells use different mechanisms to evade immune surveillance. Among these mechanisms, tumor cells express immune checkpoint inhibitor ligands and promote CD8^+^ T cell exhaustion, thus leading to the suppression of the antitumor immune response [40]. PD-1 (CD279) is an immune checkpoint inhibitor that is expressed mainly on the surface of immune effector cells, like on activated T cells, NK but also on the surface of B lymphocytes, macrophages, dendritic cells (DCs) and monocytes [41]. The level of expression of PD-1 on immune cells is low, but increases after antigen stimulation. PD-1 expression is induced on both activated CD8^+^, Tfh and Treg cells localized in the tumor microenvironment, and on activated B cells and NK cells. PD1 is also a marker of T cell activation and exhaustion [42,43,44,45].

PD-1 has two ligands: PD-L1 (B7-H1) and PD-L2 (B7-DC). Contrary to PD-1, PD-L1 is expressed at the basal level on many cell types, such as CD4^+^ and CD8^+^ T lymphocytes, tumor cells, CAFs and Tumor-associated macrophages (TAMs). Its expression can also be increased in macrophages, DCs and some activated T cells and B cells under inflammatory conditions [42,45,46]. PD-L2 is expressed on B cells, DCs and monocytes after stimulation and on a variety of tumor cells. PD-L2 binds PD-1 with a higher affinity than PD-L1 [42,46].

Among the factors responsible for PD-1, PD-L1 and PD-L2 expression, common γ chain cytokines, TLR ligands, interferons or complement have been identified. While IL-10 increases PD-1/PD-L1 expression, IL-4 and GM-CSF increase PD-L2 expression and TNFα improves PD-L1/PD-L2 expression [42]. PD-1 expression is regulated by numerous transcription factors, such as AP-1, nuclear factor of activated T cells (NFAT), NOTCH, Forkhead box protein (FOX) O1, NF-κB, STATs and IRFs [44,45]. PD-L1 and PD-L2 expression is regulated by PI3K/Akt, MAPK, NF-κB, Hedgehog, IRFs and STATs transcription factor family [42,45,46].

PD-1 is a transmembrane protein containing an extra-cellular domain to bind its ligands and an intra-cellular domain for signal transduction. The cytoplasmic part of PD-1 contains two signaling domains, immune tyrosine-based inhibition motif (ITIM) and immune tyrosine-based switch motif (ITSM). On fixation of PD-1 ligands, ITIM and ITSM tyrosine residues get phosphorylated, which enables the recruitment of Src homology region 2 domain-containing phosphatase-1 (SHP-1) and SHP-2. Recruited SHPs protein decrease TCR signaling, by inhibiting the phosphorylation of CD3ξ and ZAP-70. PD-1 ligation can also inhibit PI3K/Akt, MAPK and activates Phosphatase and tensin homolog (PTEN) pathways, thus inhibiting cell proliferation. The inhibition of these signaling pathways also dampens cytokine secretion. On activated CD8^+^ T cells, PD-1/PD-L1 ligation inhibits the secretion of IL-12, IL-10 and IFNγ. In tumor-infiltrating DCs, PD-1 signaling inhibits NF-κB-induced TNFα and IL-6 production [42,47].

Independently of PD-1, PD-L1 and PD-L2 regulate several pathways in cancer cells, such as proliferation, survival, migration and motility [48,49]. Thus, PD-L1 and PD-L2 may act as a pro-tumorigenic factor, per se. Because of the wide expression of PD-1 and PD-Ls on many cell types, their interactions, and mechanisms leading to immune tolerance are exceedingly complex. However, the widely studied mechanism is the interaction of PD-L1 on tumor cells with PD-1 on CD8^+^ T cells. The PD-1/PD-L1 pathway can be modulated by various signals in cancer cells and plays a role in maintaining immune tolerance [45]. Based on the observation that the PD-1/PD-Ls pathway drives immune escape and tumor growth, many treatments now include targeted therapy alone or in association with chemotherapy. Among these targeted therapies, anti-PD-1 antibodies (nivolumab and pembrolizumab) and anti-PD-L1 antibodies (atezolizumab, durvalumab, avelumab) have shown promising results. Despite impressive results, many patients do not respond, notably because of tumor intrinsic factors, such as cancer type, tumor mutation burden or specificities of the tumor environment [50,51]. Thus, further investigations are required to understand PD-1/PD-Ls inhibitor resistance to propose more efficient treatments based on immunotherapy to cancer patients.

Based on the above description, blocking PD-1 seems to be more effective than inhibiting PD-L1 or PD-L2, because it blunts all the pathways, as observed in non-small cell lung cancer (NSCLC) patients [52]. However, it is not quite that simple, since PD-L1 and PD-L2 can be ligands of other receptors. PD-L1 can ligate CD80 (B7-1) on APC [53]. In this context, PD-L1 blockade plus anti-CTLA-4 is more effective than anti-PD-1 plus anti-CTLA-4, because anti-PD-L1 releases CD80, which can further impact on CD28 on T cells to enable their proliferation and activation [54]. PD-L2 can interact with repulsive guidance molecule b (RGMb) expressed on naive mouse T cells, macrophages, neutrophils and dendritic cells. This entails the suppression of Th2-mediated asthma and ameliorates lung pathologies in an experimental mouse model [55]. However, the impact of PD-L2/RGMb interaction remains unknown in cancer.

## 2. Crosstalk between Inflammasomes and PD-1/PD-L1 in Cancer

Recent studies have highlighted the interest of associating anti-PD-1/L1 therapy with inhibitors or activators of inflammasome-derived cytokines (Table 1).

Among melanoma patients receiving anti-PD-1 treatment, tumor biopsies from responders present an increased “inflammasome signature” as compared to samples from progressors. Even if NLRP3, NLRP6, NLRP7, AIM2 PYCARD and CASP1 expression is increased, we cannot conclude that inflammasomes are more activated [56]. Moreover, although IL1R1 and IL18R1 are more highly expressed in responders, IL1R2, IL1RN, IL18BP (coding for IL-1 and IL-18 inhibitors) are also overexpressed in these patients. The “inflammasome signature” can be used as a marker of progression, but it cannot define whether inflammasomes, IL-1β or IL-18 are active and biologically improve anti-PD-1 therapy. Nevertheless, a correlation between NLRP3 expression and the frequency of CD8^+^ and activated memory CD4^+^ T cells was observed in responders [56].

### 2.1. NLRP3

In human head and neck squamous cell carcinoma (HNSCC), NLRP3, ASC, CASP1, IL1B and IL18 gene expression is increased as compared to oral mucosa. PDCD1 (Programmed cell death 1) and NLRP3 expression seems to be correlated. Because an increased expression of NLRP3, ASC, Caspase-1, IL-1β and IL-18 proteins is observed in tumor tissues from Tgfbr1/Pten 2cKO HNSCC mice, there is a rationale for testing the NLRP3 inflammasome inhibitor, MCC950 in this model. Thus, MCC950 is able to decrease IL-1β, regulatory immune cells (MDSCs, Treg) and exhausted PD-1^+^ CD8^+^ and CD4^+^ T cells within HNSCC tumors in mice [59]. However, the mechanism explaining how MCC950 decreases PD-1 expression in T cells, e.g., IL-1β dependency or a direct NLRP3 effect in T cells, remains to be clarified.

On the other hand, stimulation of BRAF^V600E^ PTEN^−/−^ melanoma cells (but also Lewis lung carcinoma cells) with antigen-specific CD8^+^ T cells (or with IFNγ, the cytokine produced by these cells) in the presence of anti-PD-L1 antibody, drives NLRP3 inflammasome and caspase-1 activation. These effects are not found in Pdl1^KD^ cells. Anti-PD-L1 antibody binding on PD-L1 inhibits STAT3 activation, a known inhibitor of protein kinase R (PKR). Then, PKR becomes activated and allows NLRP3 inflammasome assembly with ASC and caspase-1 activation in cancer cells. This leads to the release of HSP70 through an unknown mechanism, which binds to TLR4 in an autocrine manner. TLR4 activation induces release of CXCR2 ligands (CXCL1, CXCL2, CXCL5) by cancer cells and the recruitment of PMN-MDSCs. MDSCs are responsible for CD8^+^ T cell inhibition. This study highlighted the capacity of PD-L1 to drive immunosuppression, through NLRP3 activation in tumor cells, MDSC recruitment and CD8^+^ T cells inhibition, without exploring the effects of therapy combining anti-PD-L1 with NLRP3/caspase-1 inhibitor [57]. These findings suggest that anti-PD-L1 can also transduce a signal, i.e., by directly inducing an NLRP3 activation signal.

### 2.2. AIM2

AIM2 can regulate PD-L1 expression. When macrophages have consumed dying mammary tumor cells by phagocytosis, AIM2 senses tumor DNA, enabling caspase-1 activation and IL-1β production. Released IL-1β is able to increase PD-L1 expression in human macrophages [80]. Although the neutralization of AIM2 or IL-1β improves anti-HER2 therapy in mice, the impact of blocking this pathway on anti-PD-1/PD-L1 therapy has not been investigated.

### 2.3. Caspase-1

EG7 murine thymoma-bearing mice yield benefit from anti-PD-1 treatment. However, while this effect of immunotherapy is lost in caspase-1/11-deficient mice, Nlrp3-deficient mice still respond to this treatment [56]. Further experiments are required to determine whether other inflammasomes and/or inflammatory cytokines are implicated in this context to explain these discrepancies.

### 2.4. IL-1β

IL-1β was shown to increase PD-L1 at the membrane of cancer cells and CAFs, and associations of antibodies targeting IL-1β and PD-1 have shown promising therapeutic efficacy. BRAF^V600E^ mutation induces IL-1α/β secretion by melanoma cells, which triggers increased expression of PD-L1/L2 at the cell surface of CAFs. This expression of PD-1 ligands on CAFs is responsible for inhibition of CD8^+^ T cells in vitro. The BRAF^V600E^-specific inhibitor vemurafenib abrogates IL-1α/β secretion and restores CD8^+^ T cell function, suggesting an application of vemurafenib as an immune checkpoint regulator, through its effects on IL-1α/β production [60]. Kaposi’s sarcoma-associated herpes virus induces tumorigenesis. In the lytic viral replication phase, MAPK, NF-κB and AKT induce IL-1β expression, which is then responsible for PD-L1 expression in human iSLK.219 cells, suggesting an immune escape mechanism triggered by oncolytic viruses [61].

IL-1β can also have a direct effect on human NSCLC or gastric cancer cells, by inducing PD-L1 expression [62,63]. Moreover, recombinant IL-1β and M1 macrophage-derived IL-1β induce PD-L1 expression at the cell surface of human and murine hepatocellular carcinoma cells. This expression is made possible by the binding of p65 NF-κB/IRF1 on the PD-L1 promoter [64]. The impact of this increased expression on anti-tumor immune response remains to be investigated.

KRAS G12D transgenic mice overexpressing IL-1β in the pancreas present an increased number of PD-L1^+^ B cells in this organ, thus leading to CD8^+^ T cell anergy and pancreatic ductal adenocarcinoma (PDAC) development [66]. On the contrary, when silencing IL-1β in murine KPC pancreatic cancer cells, infiltration of orthotopic tumors by immunosuppressive cells (MDSCs, Th17, Breg, M2 macrophages) is reduced, while the number of INFγ/GzmB producing CD8^+^ T cells is increased. The establishment of this immunosuppressive tumor microenvironment by IL-1β can be explained by the fact that tumor-derived IL-1β activates pancreatic stellate cells (PSCs), which in turn shape CAFs. In this context, targeting tumor and host-derived IL-1β with anakinra improves anti-PD-1 therapy [65].

IL-1β is implicated in murine breast cancer development and metastasis, namely by favoring inflammatory immune cell infiltration in the tumor bed and CD8^+^ T cell inhibition. Moreover, the association of anti-IL-1β and anti-PD-1 mAbs inhibits tumor growth. This therapeutic improvement is associated with an increased frequency of CD8^+^ T cells intratumor [67]. The precise mechanism leading to these effect remains to be elucidated.

Il1b deficiency in mice inhibits tumor growth in different models (B16F10 and YUMM1.7 melanoma, EL4 thymoma or LLC lung cancer), while Il1r1 and Il1a deficiency in mice has no effect. Moreover, an opposing role of IL-1α and IL-1β is observed, as anti-IL-1β Ab effects are lost when added to an anti-IL-1α Ab or when used in Il1r1^−/−^ or Il1a^−/−^ mice [68]. This raises the question of using a specific anti-IL-1β Ab, such as canakinumab [21], rather than pan-anti-IL-1 Abs such as anakinra, previously and currently tested in many clinical trials [19]. The effects of IL-1β blockade are due to CD8^+^ T cells and seem to be improved in the murine EL-4 thymoma model, using anti-PD-1 Ab [68].

### 2.5. IL-18

IL-18 and PD-1/PD-L1 can regulate each other, and the use of antibodies targeting PD-1 in association either with IL-18 or anti-IL-18 has shown promising therapeutic efficacy, depending on the model.

First, PD-1 or PD-L1 blockade therapy can differentially impacts gene expression in the monocytes of patients with lung cancer. In particular, PD-L1 blockade is more prone to increase NLRP3 and IL1B as compared with anti-PD-1. Moreover, treatment of healthy donor monocyte-derived DC with anti-PD-L1 induces maturation, caspase-1 activation and IL-18 and IL-1β production, while anti-PD-1 has no effect [58]. While this study does not elucidate whether IL-18 or IL-1β induced production by anti-PD-L1 would be beneficial or detrimental for cancer treatment, it pinpoints an important discrepancy between anti-PD-1 and anti-PD-L1 mAbs.

Second, several cancer cell lines, such as murine colon (CT26), melanoma (B16F10) or human breast (MDA-MB-231) cancer cells or nasopharyngeal cancer patient tumors have been reported to secrete IL-18. Tumor-derived IL-18 induces PD-1 expression on NK cells, thus allowing neutrophils to dampen their activity through binding of PD-L1 on their membrane with PD-1 [69,70,71,72]. Pancreatic cancer cell (Panc02)-derived IL-18 was also shown to be able to increase PD-1 on B cell surface. Moreover, the association of anti-IL18 and anti-PD-1 inhibits tumor growth and metastasis in vivo [73]. The beneficial effect of combo-therapy cannot be confirmed as mono-therapy groups were not tested in this study. In the K7M2 osteosarcoma model, IL-18 seems to be implicated in MDSC recruitment at the tumor site and the association of anti-PD-1 with IL-18BP (an inhibitor of IL-18) increases the proportion of CD8^+^ IFNγ^+^ GzmB^+^ T cell tumor infiltration, and decreases tumor weight [81].

CD39 belongs to the ectonucleotidase family, responsible for the degradation of ATP into ADP/AMP [82]. Thus, inhibiting CD39 enables greater NLRP3 inflammasome activation and IL-1β and IL-18 maturation. In different murine cancer models, the association of blocking Abs against CD39 and PD-1 slows tumor growth through a NLRP3/IL-18-dependent and IL-1β-independent mechanism [74,75]. This may be explained by the capacity of IL-18 within the tumor to turn “cold” resistant tumors (high macrophages) into “hot” tumors (high CD8^+^/IFNγ^+^/PD-1^+^ T cells) [75].

IL-18 also enhances anti-PD1 antitumor effect in murine colon cancer (CT26) or melanoma (B16F10) peritoneal dissemination models in a NK and CD8^+^ T cell-dependent manner. This effect is not found in a breast cancer (4T1) peritoneal dissemination model [76]. In the same way, IL-18 has no effect on tumor growth in murine MC-38 colon cancer models. Consequently, the authors designed DR-18, an IL-18 resistant to IL-18BP inhibition, which enhances tumor regression. This effect was improved when DR-18 was associated with anti-PD-1. The effects of DR-18 rely on the polarization of CD8^+^ T cells into stem-like precursor cells (CD8^+^, PD-1^+^, TIM-3^+^, TCF1^+^, TOX^−^) and NK cell maturation [77].

Taken together, these studies highlight the fact that IL-18 may improve immunotherapy only in some cases, and that it depends on the tumor immune-infiltrating cells and their capacity to secrete inhibitory molecules, such as IL-18BP. 

### 2.6. IL-33

IL-33 has been shown to regulate PD-1/PD-L1 expression and to improve anti-PD-1 therapy. Tc9 cells are a subtype of CD8^+^ T cells generated in the presence of TGFβ and IL-4, with antitumor properties. IL-33 (alone or in co-culture with bone marrow-derived DCs) increases Tc9 activation (IL-9, GzmB, proliferation) and decreases PD-1 expression at the cell surface. In bone marrow-derived DC cell transfer or Tc9 cell transfer therapeutic models, IL-33 injection delays tumor growth [83]. However, the impact of blocking PD-1 was not investigated. In acute myeloid leukemia (AML)-bearing mice, IL-33 also increases CD8^+^ T cell proliferation and activation, and reverses DC tolerance. IL-33 is also able to increase PD-1 and PD-L1 expression at the cell surface of CD8^+^ T cells and tumor cells respectively. Thus, combining IL-33 with an anti-PD-1 Ab yields a more substantial antitumor effect than monotherapy [79]. Invalidating ST2 in mice or IL-33 in MC-38 colon cancer cells impedes anti-tumor effects of anti-PD-1 mAb. Moreover, IL-33-overexpressing B16 melanoma cells have slower growth than WT cells, and the use of anti-PD-1 mAb improves this delay of tumor growth. The importance of IL-33 was explained by its capacity to increase the percentage of CD103^+^ DCs and CD8^+^ CD103^+^ T cells within the tumor, a sub-population important in the killing of malignant cells [84].

Innate lymphoid cells 2 (ILC2) express ST2 on their membrane and respond to IL-33 (proliferation, IL-13 secretion). IL-33 also increases PD-1 (and PD-L1, but not in all studies) expression in these cells, which then participate in the inhibition of uncontrolled ILC2 proliferation/activation (IL-5, IL-13, IL-9 secretion and GATA3 expression) [85,86,87]. In an orthotopic PDAC model, IL-33 drives ILC2 to enable tissue-specific cancer immunity, namely by increasing intratumor activated CD8^+^ T cells and DCs. Anti-PD-1 mAb exacerbates tumor infiltrating ILC2 and then amplifies this phenomenon. These results were not replicated in a subcutaneous PDAC model. This discrepancy was explained by the fact that in this latter model, ILC2 expressed IL-18R1, and not ST2, and IL-18 played a similar role in this model to IL-33 in the orthotopic model [78]. Moreover, in several lung metastasis models, IL-33-activated ILC2 suppress NK cells. Targeting IL-33 removes NK cell suppression and reduces tumor burden [88]. Thus, depending on the cancer models used, IL-33 may or may not improve anti-PD-1 therapy.

## 3. Crosstalk between Inflammasomes and PD-1/PD-L1 Outside the Setting of Cancer

PD-1/PDL-1 and inflammasome-derived cytokines have been shown to be linked in in vitro experiments and to play important roles in diseases other than cancer.

### 3.1. In Vitro Observations

IL-1β and PD-1/PD-L1 interconnections have been studied in different models in vitro. PD-L1 blockage increases the capacity of M2 macrophages to produce IL-1β after LPS treatment [89]. The effect of anti-PD-L1 alone was not investigated.

On the other hand, myeloid cells collected from the peritoneal cavity of Il1r1^−/−^ mice express higher levels of PD-L1 than WT mice, pleading for a role of IL-1α and/or IL-1β in the regulation of PD-L1 expression [90]. TLR4 or TLR7/8 stimulation induces PD-L1 and PD-L2 expression on human monocyte-derived DCs. Divergent observations have been made regarding the role of IL-1β in this context. In one study, IL-1β (100 to 1000ng/mL) was responsible for TLR-mediated effects and could directly induce PD-L1 expression, while in another study (IL-1β: 30ng/mL), it appeared not to be implicated [91,92]. These discrepancies may be explained by the differences in IL-1β concentrations used.

IL-1β was shown to increase PD-L1 expression in other cell types. In human periodontal ligament stem cells (hPDLSCs), while TNFα and IFNγ heighten both PD-L1 and PD-L2 expression, IL-1β only increases PD-L1 expression [93,94]. IL-1β also induced PD-L1 expression at the cell surface of human placenta-derived mesenchymal stromal cells in an NF-κB and JAK1/2-dependent manner [95]. However, the physiological impact of IL-1β-mediated PD-L1 expression remains unknown. In co-culture experiments, Treg maintain PD-1 expression on NK cells and suppress their immune functions (IFNγ production and proliferation). Blocking IL-37 or its receptor IL-1R8 restores NK cell functions and decreases PD-1 expression. On the contrary, adding IL-37 increases PD-1 expression on NK cells [96]. Thus, the importance of targeting IL-37 might be explored in cancer treatment.

### 3.2. PD-1/PD-L1 and Inflammasome Interactions in Other Diseases

In infectious diseases, PD-1 influences inflammasome-processed cytokines. In a murine tuberculosis model, PD-1 inhibition induces an increase in cytokines such as IL-1, IL-6 and IL-17 after 22 days of infection [97]. In LPS-mediated endotoxemia, PD-1-deficient mice have increased serum levels of IL-1β, TNFα, IL-12 and IL-17 and a lower survival rate as compared with WT mice, suggesting that PD-1 can control inflammatory cytokine production [98]. Using hypoxia to mimic pulmonary hypertension, STAT1 is activated and then transduces the expression of PD-L1 in smooth muscle cells. Increased PD-L1 expression is responsible for the induction of pyroptosis-mediated fibrosis [99]. While PD-L1 was shown here to regulate caspase-1 activation and IL-18 maturation, the precise mechanism leading to PD-L1-mediated pyroptotic cell death remains to be elucidated. On the contrary, caspase-1 seems to be able to regulate PD-L1 expression. As compared to WT, APCs from Caspase-1,11-RipK3 deficient mice highly express PD-L1, which is correlated with the expression of PD-1 on primed CD8^+^ T cells [100].

Targeting PD-1/PD-L1 can lead to Checkpoint inhibitor pneumonitis (CIP). In a cohort involving several types of cancer patients, anti-PD-1 or anti-PD-L1-mediated CIP is associated with an abnormal number of IL-1β-containing monocytes. Surprisingly, IL-1β is not found in bronchoalveolar fluids, raising the question of whether IL-1β is overexpressed and/or sequestrated in monocytes, and/or released at earlier timepoints than those studied, after anti-PD-1/PD-L1 therapy [101].

IL-1β was proposed to have opposite effects on the PD-1/PD-L1 pathway. *Streptococcus pyogenes*-derived exotoxin A induces IL-1β production by primary monocytes, which in turn increases PD-L1 expression in an autocrine way. In co-culture experiments, these treated monocytes are able to increase Treg proportion among CD4^+^ T cells [102]. In vitro, IL-1β can counteract the suppressive effects of PD-1 ligation on CD4^+^ T cells from healthy donors, but not from patients with rheumatoid arthritis or psoriatic arthritis. These diseases are associated with high levels of TNFα, IL-6 and IL-1β, suggesting that these inflammatory cytokines dampen PD-1/PD-L1 signaling. While this might be explained by the capacity of IL-6 and TNFα to induce soluble PD-1 secretion, no mechanism was proposed for IL-1β [103].

IL-18 (in association with IL-2) increases PD-L1 expression on murine NK cells. Blockade of PD-L1 almost completely abrogated the killing of activated CTL by IL-18-stimulated NK cells in vitro. When injected to streptozotocin-treated mice (which present hyperglycemia), these NK cells lowered blood glucose level, a hallmark of diabetes [104]. Epithelial damage caused by helminths or allergens leads to IL-33 release, which in turn favors PD-L1 expression on ILC2. Then, PD-L1 interacts with PD-1 localized on CD4^+^ T cells, enabling their polarization into Th2 cells (Gata3 expression and IL-13 secretion) and worm expulsion by epithelial cells [105]. Similarly, several reports highlight the crosstalk between IL-33 and PD-1 in the regulation of immune responses to pathogens [85], allergic asthma [86], or obesity [87]. In vivo, IL-33 polarizes macrophages towards an M2/PD-L2^+^ phenotype, which, when injected into experimental autoimmune encephalomyelitis (EAE) mice, can attenuate disease development [106]. Thus, investigations in inflammatory diseases propose crosstalk between PD-1/PD-L1 and inflammasomes that should be adapted in the context of cancer.

## 4. Conclusions

We have reported here that the crosstalk between inflammasomes, related cytokines and the PD-1/PD-L1/2 pathway is highly complex (Table 1). Although inhibiting IL-1β or IL-18 in combination with anti-PD-1/L1 has shown promising therapeutic effects in some pre-clinical models, the means to inhibit these cytokines must be questioned. To block production of both cytokines, inflammasome inhibitors could be used. However, the inflammasome implicated in different cancers must first be known in order to be specifically targeted. The other means would be to target caspase-1. To the best of our knowledge, the use of a specific caspase-1 inhibitor in the clinical setting is not on the cards. Moreover, inhibiting inflammasome/caspase-1 would lead to decreased production of IL-1β and IL-18, but perhaps also to a lack of IL-33 cleavage. As IL-33 seems to cooperate with anti-PD-1, it would be doubly beneficial. Nevertheless, IL-18 can also improve anti-PD-1 therapy in some preclinical models. Thus, targeting one cytokine specifically according to the cancer profile is the widely proposed plan of action. This can be considered by studying cytokines or their receptor expression. This idea was strengthened by showing that using a similar PDAC model, the cytokine required, i.e., IL-18 or IL-33, depends on the site of injection, that is to say the tumor microenvironment. Moreover, cytokine gene expression should be viewed with caution, as IL1B and IL18 expression do not reflect the bioactive form content, and do not take into consideration the presence of inhibitory molecules, such as IL-1RA or IL-18BP. To target IL-1β, canakinumab and anakinra are mostly used in many clinical trials. While the first specifically inhibits IL-1β, the latter inhibits both IL-1α and β. Thus, the importance of IL-1α should be considered before using such antibodies. Nevertheless, four ongoing clinical trials can be found on ClinicalTrial.gov at that moment (Table 2).

To improve our knowledge of the capacity of inflammasomes to modulate PD-1/L1/L2 expression, the impact of NLRs and associated cytokines on transcription factors controlling their expression must be studied, as was shown for IL-1β-mediated p65 NF-κB/IRF1 fixation on PD-L1 promoters. In addition, the impact of PD-1/L1/L2 on transcription factors controlling inflammasome constituent expression and/or caspase-1 activation should be considered.

To conclude, many relatively new studies have begun to decipher the importance of targeting PD-1/L1/L2 in association either with specific antibodies targeting inflammatory cytokines or with recombinant cytokines. However, further research is required to fully understand how and when inflammatory cytokines should be targeted in order to improve immune checkpoint therapy.

## Figures and Tables

**Table 1:** Effects of targeting PD-1/L1 or inflammasome-associated molecules in cancer.

	Target	Increased Effects	Decreased Effects	Cancer Models	Combo Therapy Effects	Ref.
**Inhibition**	PD-L1	NLRP3 inflammasome and caspase-1		BRAF^V600E^ PTEN^−/−^ melanoma cells	↘ tumor	[57]
	PD-L1	NLRP3 and IL1B IL-18 and IL-1β production			Not tested	[58]
	NLRP3		MDSCs, Treg, PD-1^+^ CD8^+^ and CD4^+^ T cells	Tgfbr1/Pten 2cKO HNSCC	Not tested	[59]
	AIM2/ IL-1β		PD-L1	Human breast cancer cells	Not tested	[55]
	Caspase-1			EG7 murine thymoma	↗ tumor	[56]
	IL-1β		PD-L1/L2	Human BRAF^V600E^ melanoma	Not tested	[60]
	IL-1β		PD-L1	iSLK.219 Kaposi’s sarcoma	Not tested	[61]
	IL-1β		PD-L1	Human NSCLC or gastric cancer cells	Not tested	[62,63]
	IL-1β		PD-L1	Human and murine hepatocellular carcinoma cells	Not tested	[64]
	IL-1β	INFγ/GzmB producing CD8^+^ T cells	MDSCs, Th17, Breg, M2 macrophages	Orthotopic murine KPC pancreatic cancer cells	↘ tumor	[65]
	IL-1β		PD-L1	KRAS G12D transgenic mice overexpressing IL-1β in the pancreas	Not tested	[66]
	IL-1β/ PD-1	CD8^+^ T		Murine breast cancer	↘ tumor	[67]
	IL-1β	CD8^+^ T cells		EL-4 thymoma	↘ tumor	[68]
	IL-18		PD-1	Murine colon (CT26), melanoma (B16F10) or human breast (MDA-MB-231) cancer cells or nasopharyngeal cancer patient tumors	Not tested	[69,70,71,72]
	IL-18		PD-1	Murine pancreatic cancer cell (Panc02)	↘ tumor	[73]
	CD39/ PD-1			4T1.2 mammary carcinoma-induced metastasis	↘ tumor	[74]
	CD39	IL-18, CD8^+^/IFNγ^+^/PD-1^+^ T cells	macrophages	Murine SM1WT1, B16F10 melanoma and RM-1 prostate carcinoma	↘ tumor	[75]
**Recombinant proteins**	IL-18			Murine colon cancer (CT26), melanoma (B16F10) peritoneal dissemination models	↘ tumor	[76]
	Resistant IL-18 (DR-18)	CD8^+^/PD-1^+^ and NK cell maturation		Murine MC-38 colon cancer	↘ tumor	[77]
	IL-18	ILC2		Subcutaneous PDAC	↘ tumor	[78]
	IL-33	CD8^+^ T cells and DCs		Orthotopic PDAC	↘tumor	[78]
	IL-33	PD-1/L1, CD8 T cell proliferation and activation		Murine AML C1498.SIY cells	↘ tumor	[79]

**Table 2:** Ongoing clinical trials targeting IL-1 and PD-1/PDL-1 pathways.

Id	Cancer Type	Study Phase	Target 1 (Inhibitor)	Target 2 (Inhibitor)	Other Treatments
NCT03631199	NSCLC	III	IL-1β (canakinumab)	PD-1 (pembrolizumab)	Carboplatin, cisplatin, paclitaxel, nab-paclitaxel, pemetrexed
NCT04121442	Solid tumors	I/IIa	IL1R1 (Isunakinra)	PD-1 or PD-L1	Not specified
NCT04452214	NSCLC, urothelial, malignant melanoma, HNSCC	1b	IL-1RAcP (CAN04)	PD-1 (pembrolizumab)	Not specified
NCT04581343	Metastatic PDAC	1B	IL-1β (canakinumab)	PD-1 (spartalizumab (PDR001))	Gemcitabine, nab-paclitaxel
